# Effect of low oxygen stress on the metabolic responses of tomato fruit cells

**DOI:** 10.1016/j.heliyon.2024.e24566

**Published:** 2024-01-24

**Authors:** Md. Sultan Mahomud, Md. Nahidul Islam, Joysree Roy

**Affiliations:** a Department of Food Engineering and Technology, Hajee Mohammad Danesh Science and Technology University, Dinajpur, 5200, Bangladesh; b Department of Agro-Processing, Bangabandhu Sheikh Mujibur Rahman Agricultural University, Gazipur, 1706, Bangladesh; c Institute of Food Safety and Processing, Bangabandhu Sheikh Mujibur Rahman Agricultural University, Gazipur, 1706, Bangladesh

**Keywords:** Tomato fruit, Cell culture, Hypoxic stress, Anoxic stress, Metabolomics

## Abstract

Postharvest losses of fruits and vegetables can occur due to cell breakdown and browning during controlled atmosphere storage as a result of low oxygen (O_2_) stress. Therefore, the study was designed to better understand the underlying mechanisms of the response of isolated tomato fruit cells incubated at low O_2_ (hypoxic and anoxic) conditions as a model system. The O_2_ stress conditions used for the experiment were based on the results of the Michaelis–Menten constant (*K*_*m*_) of respiration. A total of 56 polar metabolites belonging mainly to different functional groups, including amino acids, organic acids, sugars and sugar alcohols, were identified using GC-MS. O_2_ stress stimulated the biosynthesis of most of the free amino acids while decreasing the synthesis of most of the organic acids (especially those linked to the tricarboxylic acid cycle), sugars (except for ribose) and other nitrogen-containing compounds. The down-regulation of these TCA cycle metabolites served to provide energy to ensure the survival of the cell. Increases in the sugar alcohol levels and induction of fermentative metabolism were observed under low O_2_ stress. By employing multivariate statistics, metabolites were identified that were essential to the oxygen stress response and establishing the correlation between metabolite abundance, oxygen levels, and incubation period were achievable. A higher correlation was observed between the O_2_ levels and most of the metabolites.

## 1 Introduction

Plants are sessile and developed a protective and/or adaptive mechanism against adverse environmental conditions such as too-extreme temperatures, salinity, drought, etc., due to their physiological homeostasis [1,2]. To ensure and maintain this homeostasis, the plant's central carbon metabolism has to be very flexible [3]. Oxygen stress is a common environmental phenomenon that has an impact on central metabolic pathways [4]. In order for higher plants to have a metabolic processes, oxygen is absolutely necessary [5]. However, plants can be exposed to a wide range of oxygen concentration in natural or experimental settings, from normal levels (normoxic) to shortage (hypoxia) to complete absence (anoxia). According to Drew [6], when oxidative phosphorylation is not inhibited by the availability of oxygen, normoxic conditions predominate. In hypoxia, the partial pressure of O_2_ (PPO) is low enough to restrict the amount of ATP that cell can produce, whereas anoxia is reached when the amount of ATP produced by cell is negligible in comparison to the amount produced by fermentation and glycolysis [7]. In addition, it produces reactive oxygen species (ROS) that damage the cell of a plant. Normally, these ROS are produced in all living plants during metabolism. Plant cells adapt to oxidative stress by rewiring their metabolic networks to either compensate for enzyme damage or to facilitate adaptive responses [8].

The potential of metabolomics for elucidating the response-to-stress mechanisms, managing post-harvest storage as well as develop resistance strategies in affected cultivars have recently been reviewed by Carrera, Noceda [9]. Using metabolomic approach, the response of dragon fruit under controlled atmosphere storage has been investigated by Ho, Tran [10]. The effects of low PPO on metabolism of three apple varieties have been recently investigated by Park, Al Shoffe [11]. In addition to its culinary and nutritional benefits, tomatoes are an economically significant crop, growing all over the world – in outdoor fields, greenhouses and net houses [12]. After white potatoes, it is the most extensively cultivated vegetable crop all over the world [13]. Moreover, it is characterized by dramatic metabolic changes [14]. Storing tomatoes at low levels of O_2_ can induce oxidative stress that leads to a wide range of metabolic changes. At extreme O_2_ stress, aerobic respiration is shifted to fermentative metabolism, which can lead to off-flavor and quality loss, resulting in high economic losses. Ampofo‐Asiama, Baiye [15] studied the oxygen stress on tomato fruit cell at 0, 1 and 21 kPa using ^13^C level in the media. To gain better insight into the mechanisms underlying the response of isolated tomato cells when incubated under controlled conditions of low oxygen (hypoxic and anoxic) based on their *K*_*m*_ values, this study was conducted without the use of ^13^C level in the media. In addition, to facilitate the design of the controlled atmosphere for reducing post-harvest losses of tomato, metabolites were evaluated using gas chromatography-mass spectroscopy based metabolic profiling which were actively involved in the response of oxidative stress.

## 2 Materials and methods

### 2.1 Collection of tomatoes

Viable tomato pericarp cells were isolated from the pericarp tissues of ripened tomatoes (*Lycopersicum esculentum*var *Admiro*) obtained from a greenhouse (Gert Reijnders Tomato Greenhouse). The tomatoes were picked at the ripening stage and then weighed, sorted and grouped according to their physiological age. This physiological grouping was based on fruit color measurements at the equator of the fruits in the laboratory with a CM-2500 d spectrophotometer (Konica Minolta, Tokyo, Japan) calibrated against a red reference plate, as shown by Oms-Oliu, Hertog [16].

### 2.2 Tomato cell isolation and viability test

#### 2.2.1 Isolation (pectinase) and wash buffer preparation

The isolation (pectinase) of buffer consisted of 3 mM MES (4-Morpholineethanesulfonic acid) sodium salt at a pH of 5.8, supplemented with 150 mM glucose, 4.3 g/L Murashige and Skoog basal salt mixture (Sigma, Diegem, Belgium), 7 mM calcium nitrate tertrahydrate, 0.1 % bovine serum albumin (BSA) and 0.1 % Macerozyme (R-10) pectinase enzyme. A wash buffer was also prepared consisting of the above-mentioned chemicals, with an additional R-10 macerozyme enzyme [17]. For the activation of the pectinase, proteases and lipases, 50 mL of pectinase buffer was poured into Schott duran bottles, heated for 10 min at 55 °C in a water bath and immediately transferred on ice to be cooled to room temperature.

#### 2.2.2 Sample preparation and digestion

Sample preparation and digestion was carried out according to the method of Castoria, Mannina [18] with little modification. Ripe tomatoes were cut in their longitudinal sections. Seeds, skin and placenta were removed, and the pericarp tissues were chopped carefully into small blocks of tissues to increase the isolation efficiency of the enzyme and then washed to remove the broken cell debris. 50 g of the chopped tomato blocks were submerged in duran bottles and incubated for 2 h at 20 °C with gentle shaking for the release of the cells. The isolated tomato cells were then separated from the tissue pieces by filtration, washed with the enzyme-free buffer to remove the enzyme and cell debris, and then re-suspended in the enzyme-free buffer.

#### 2.2.3 Viability measurement

The viability measurement of tomato calls was performed by direct microscopic count with Evans blue exclusion test based on Puschmann and Romani [19]. In a 2 mL Eppendorf tube containing 500 μL of cell suspension, two drops of 0.5 % (w/v) Evans blue solution were added and gently mixed. 50 μL mixed cell suspension was smeared onto a microscopic slide and then observed by binocular microscope to count both the living and dead cells.

#### 2.2.4 Tomato cells respiration measurement

The oxygen uptake rate of the isolated tomato cells was measured using Clark's type oxygen electrode that was set up at the bottom of a small biological oxygen monitoring (BOM) device with a maximal volume of 5 mL [20]. The medium and cell suspension was poured into the incubation chamber of the BOM, and after flushing the cell medium with 21 kPa of oxygen for 5 min, the headspace of the BOM was tightened with a screw top (headspace reducer). The temperature in the chamber was kept stable at 20 °C with a water jacket. A data logger (Agilent Technologies, Belgium) was connected to the controller of the BOM device for recording the data [21]. Following the bubble-up and calibration of the cell suspension with a gas mixture, the media’s oxygen depletion was monitored. Consequent current flowing in between conductors is directly proportional to the device’s PPO. Three replicate experiments were carried out to measure the respiration characteristics, and the result of changes in abiotic stresses on the dissolving capacity of O_2_ in liquid media was adjusted [22].

#### 2.2.5 Experimental design in bioreactor

During the first 15 min, 400 mL of tomato cells that had been isolated were moved to a bioreactor (Lambda Minifor, Lambda Laboratory Instruments, Brno, Czech Republic) that was kept at a temperature of 20 °C. Gas was bubbled through the media at a speed of ten L per hour to achieve varied O2 concentrations (21 kPa, 5 kPa, and 0 kPa), with the medium's pH being maintained at 5.8 all the time using a water bath. These O_2_ levels were selected for the experiment based on the results of the Michaelis-Menten constant (K_m_) of respiration. Prior to further investigation, cells were collected (about 10 mL of medium containing cells) for 0, 0.5, 1, 1.5, 2, 3, 4, 5, and 6 h, snap-frozen in liquid nitrogen, and kept at −80 °C.

#### 2.2.6 GC-MS analysis

Sample preparation, extraction and derivatization prior to GC-MS analysis were carried out as described by Oms-Oliu, Hertog [16] and Roessner, Wagner [23] with few modifications. The frozen tomato cell samples were freeze-dried overnight to a constant weight and then weighed in Eppendorf tubes prior to extraction and derivatization. 10 mg of lyophilized tomato samples were extracted at 70 °C for 15 min in a thermomixer using 700 μL of CH_3_OH that includes 45 μL of an internal standard (phenyl-beta-d-glucopyranoside in CH_3_OH) with a concentration of 291 ng/μL. The polar fraction was separated from the non-polar fraction by adding 325 μL of CHCl_3_ after vigorously vortexing 700 μL of water. After spinning the combination in a centrifuge for 5 min at 14,000 rpm, 500 mL of the resulting polar liquid was removed and dried for 1 h under a nitrogen stream. Oximation of the dehydrated residue was carried out by the addition of 40 μL of 20 mg/mL CH_3_ONH_2_·HCl (Sigma, Belgium) in C_5_H_5_N, vortexed for 10 s and kept in a controlled environment at 37° Celsius for 1.5 h. A thermomixer was used to conduct trimethylsilylation at 37 °C for 30 min following the addition of 60 μL C_6_H_12_F_3_NOSi (BSTFA). The gas-chromatography column was thereafter filled with 1 μL of sample volume (GC 7890A, Agilent Technologies, The USA).

#### 2.2.7 Metabolites separation, identification and quantification

All samples were run twice in the GC-MS: initially, a split mode injection of 1 μL of sample was performed with a split ratio of 1:10 in order to measure the very abundant chemicals, especially sugars and then 1 μL was injected in pulsed-splitless mode to determine the less abundant molecules mainly the organic acid, amino acid and fatty acid. Inside the region of overloaded peaks, the mass detector was turned off for less concentrated substances. The gas chromatographic separation was performed on a 30 m long HP-5MS column with 0.25 mm inner diameter and 0.25 μm film thickness (Agilent Technologies, 122 Palo Alto, CA, USA). As the carrier, helium gas was used in a steady stream of 1 ml/min. The analysis was performed in a GC oven using program as follows: 50 °C for 1 min of isothermal heating, then a ramp of 10 °C per minute to 310 °C, and finally, 1 min of heating at 310 °C. The temp of the source of ions was changed to 230 °C, while the sample input and interface temperatures were adjusted to 230 °C and 250 °C, respectively [24].

By matching retention durations and MS spectra with an internal library and verifying with Agilent, Fiehn GC-MS Metabolomics Retention Time Locking library (Agilent Technologies Inc., Wilmington, USA), metabolites were identified. For comparative analysis, the peak areas of the compounds were normalized using the weight of the dried samples, the number of live cells and the peak area of the C_12_H_16_O_6_ (internal standard) to correct within each chromatogram; as a result, the relative abundance ratio for all metabolites was achieved [25].

#### 2.2.8 Statistical analysis

Using R statistical software (Version 3.1.0, R Foundation, Vienna, Austria), univariate statistical test was conducted to analyze the variation among the levels of the metabolites under the four oxygen levels at the various time points. The *t*-test with a significance level of p = 0.05 was used to determine the difference among mean values. The Unscrambler (version 10.2, CAMO ASA, Norway) was used for chemometric analysis using a well-known partial least square discriminate analysis (PLS-DA) [26], in order to discriminate and identify which metabolites concentration changes significantly in response to increasing oxygen levels from anoxic to normoxic conditions. The abundances of the metabolites were designated as X-variables in the PLS-DA, whereas the various O_2_ levels were regarded as continuous Y-variables. The variables had equal variance and the data was mean-centered [27].

## 3 Results and discussion

### 3.1 Viability testing of tomato fruit cells

**Fig. 1:**
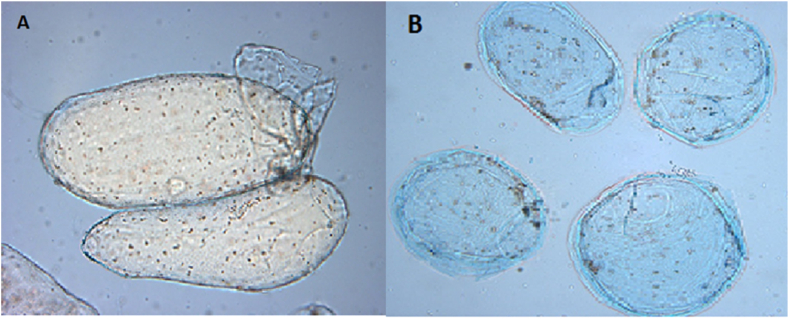
Picture of a viable (A) and a dead (B) cell isolated from intact tomato tissues. Viable tomato cells were capable of excluding Evan's blue dye.

Initially, the number of living organisms was about 85 % and decreased slowly to about 60 % after 6 h. Living and death cells were distinguished by observing the blue color as shown in Fig. 1(A and B). Living cells excluded Evan's blue dye by retaining their membrane integrity (semi-permeability) and looked white against a blue background, while dead cells lost their membrane integrity as a result of broken cell walls and, thus, did not exclude the Evans blue dye. Evans blue exclusion method has been widely used in testing the viability of many plant samples. With this method, the viability of protoplast and cell suspensions of *Coffeaarabica*cv. Catimor were found to be 93.8 % and 53 %, respectively [28]. Steward, Martin [29] found 80 % of the viability of the cell suspension culture of Alfalfa *(Medicago sativa* L.) which was grown in a bioreactor using a batch procedure. Moreover, Smith, Reider [30] showed that dead cells increased with decreasing the number of living cells during the senescence of plant tissue cultures.

### 3.2 Tomato cell's respiration characteristics

**Fig. 2:**
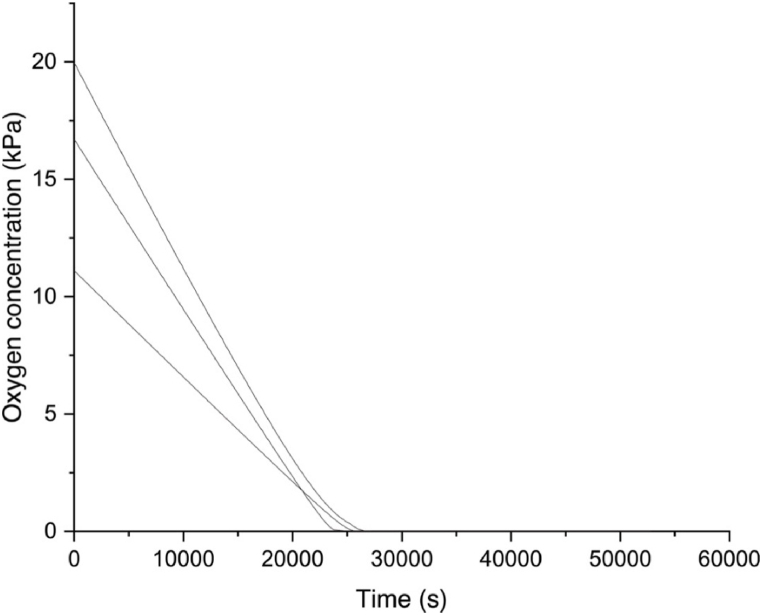
Oxygen depletion curve for tomato cells at 20 °C.

**Fig. 3:**
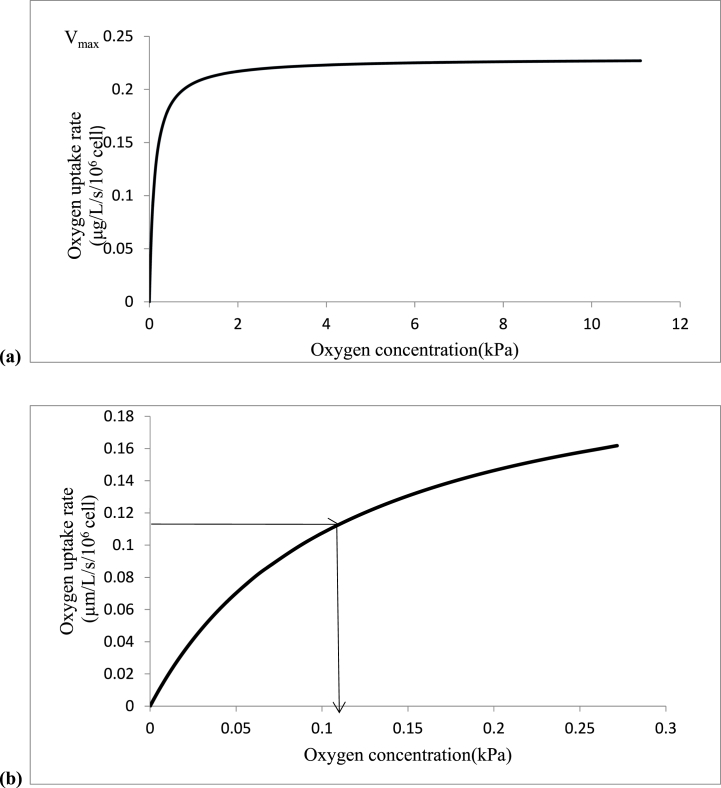
Oxygen uptake rates of tomato fruit cells (a) and magnification of the curve to read the *K*_*m*_ value (b).

The oxygen concentration at which half of the maximum oxygen uptake rate is reached, is *K*_*m*_, which is the parameter that has been used to characterize the effect of oxygen concentration on oxygen uptake rate. The BOM's oxygen depletion was measured to determine this *K*_*m*_ of respiration (Fig. 2), and equation (1) was used to compute the oxygen uptake rateand presented in Fig. 3 (a,b).

(1) $$\frac{d\left[O_{2}\right]}{dt}=V_{\max}\frac{n.\left[O_{2}\right]}{K_{m}+\left[O_{2}\right]}$$

where *V*_max_ is the maximum O_2_ uptake rate [μg/(L x s x 10^6^ cells)],*n* is the number of cells (10^6^ cells) and *K*_*m*_ = Michaelis-Menten constant.

A *K*_*m*_ of 3.54 μM equivalent to 0.12 kPa of oxygen concentration and a V_max_ of 0.23 μg/(L/s/10^6^ cells) was obtained (Fig. 3 -a,b). In this research, three oxygen values were selected based on the *K*_*m*_ of respiration: a concentration of oxygen that is below zeor, above five, and twenty one kPa (control). This minimum oxygen concentration of 0.12 kPa (when O_2_ is below 0.2 %) corresponds to anaerobic respiration in plant cells [31].

Different values of *K*_*m*_ for different fruits can be found in the literature. Lammertyn, Franck [32] calculated that conference pear cell protoplasts in suspension have a Km value of 3 μM. Solomos [33] discovered that the isolated cytochrome-c-oxidase in apples had a Km value of 0.1 μM. In isolated mitochondria of soybean, depending on the organ, *K*_*m*_ values for the alternative pathways were determined to be 9.9, 2.5, 1.6 and 1.8 μM as well as 0.05, 0.125 and 0.147 μM for cytochrome *c* oxidase were estimated [[34], [35], [36]]. The *K*_*m*_ value of pear fruits stored under controlled atmosphere conditions was >1 μM while that of ripe fruits was <0.1 μM with external PPO of 2.5 kPa [37]. The estimated *K*_*m*_ value of this experiment was a little bit higher than others because of intercellular gas diffusion [32].

### 3.3 Identification of tomato fruit metabolites

In total, 56 metabolites were identified from the chromatogram. In tomato leaves, Schauer [38] identified 64 metabolites, while 22 amino acids, 7 sugar and 6 organic acids were quantified in the tomato leaf and green and red fruit by Roessner-Tunali, Hegemann [39]. On the other hand, in tubers, 180 polar and/or non - polar metabolites were discovered [40]. Lehmann, Schwarzländer [41] identified 56 metabolites of Arabidopsis roots to oxidative stress by GC-MS. In conference pears, 64 metabolites were identified by Pedreschi, Franck [42] and 34 metabolites were identified by Franck, Lammerteyn [43].

### 3.4 Changes in metabolome levels during incubation to low oxygen stress

The generation of oxygen stress had an impact on the metabolic profile of tomato cells that were isolated. Tentatively, 56 metabolites from the proportion of polar metabolites were discovered, quantified and normalized relative to the starting values (0 h) in isolated tomato cells (Fig. 4a, Fig. 4b). Different concentrations of dissolved O_2_ in the tomato cells media in the bioreactor lead to distinct amounts of the majority of primary metabolites (Fig. 4a, Fig. 4b).

**Fig. 4a:**
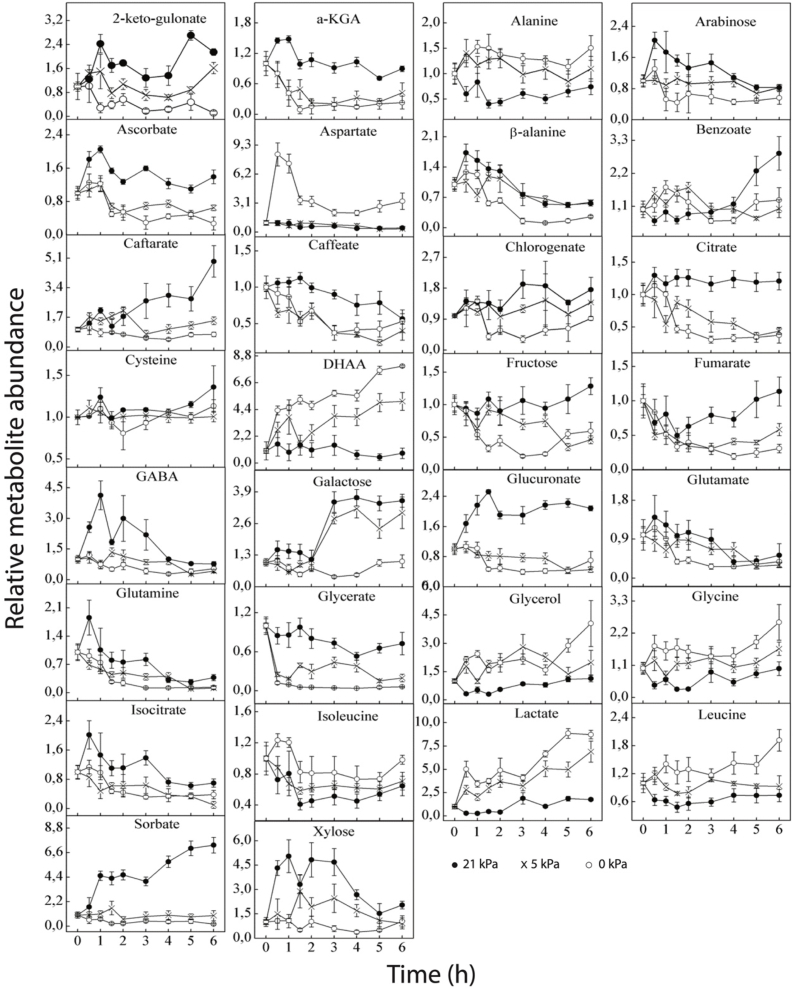
Fig. 4 is divided into two parts. This part shows the changes in the abundance of 30 metabolites in isolated tomato cells following the introduction of low O_2_ stress. All values were the average of three replicates, with the error bars indicating the standard error of the mean. Metabolite levels were expressed relative to the starting values.

**Fig. 4b:**
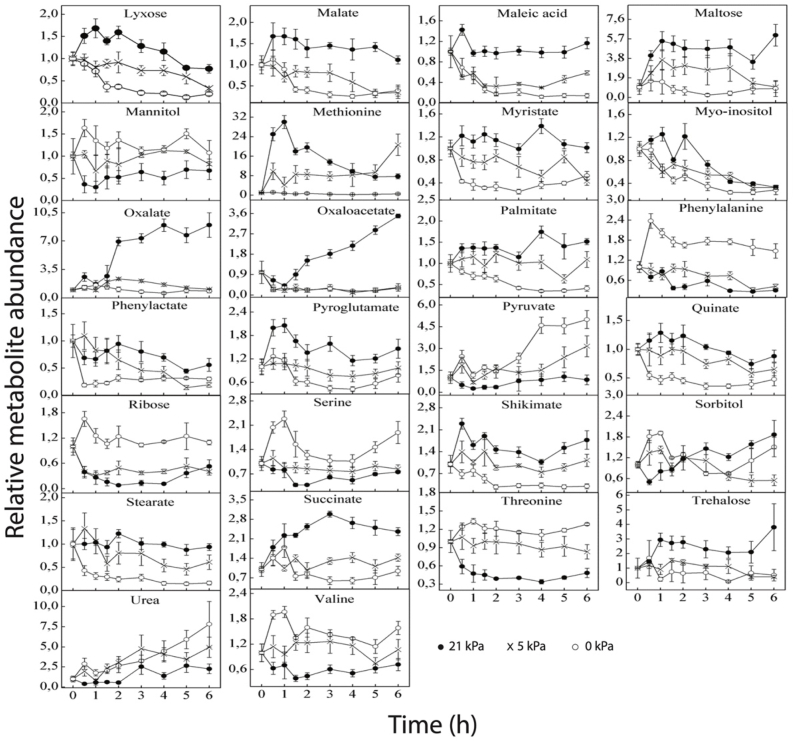
This part of Fig. 4, shows changes in the abundance of another 26 metabolites in isolated tomato cells following the introduction of low O_2_ stress. All values were the average of three replicates, with the error bars indicating the standard error of the mean. Metabolite levels were expressed relative to the starting values.

**Fig. 5:**
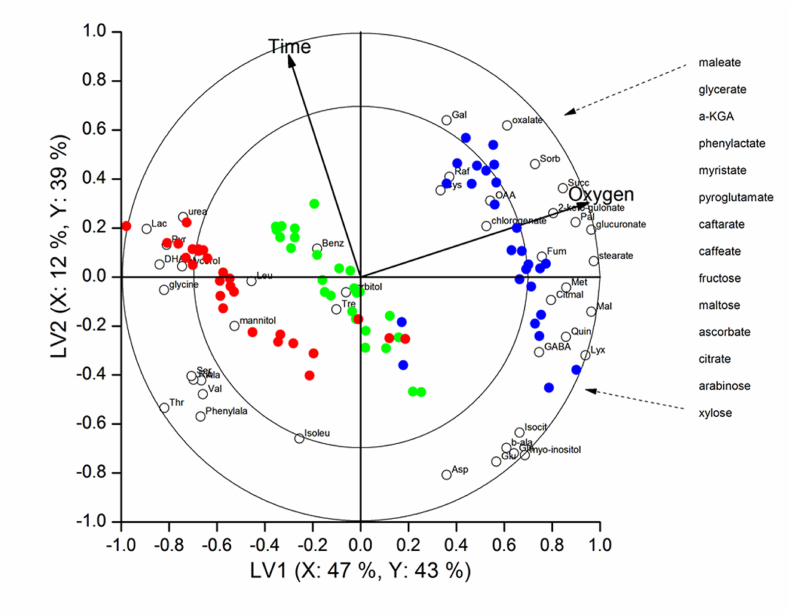
PLS-DA plots of tomato fruit cells representing different O_2_ conditions with time (LV1 versus LV2). Red circle: 0 kPa; green circle: 5 kPa; and blue circle: 21 kPa. The percentage explained variances were indicated in the axes.

PLS-DA was performed in order to sharpen the effects between the different oxygen levels on tomato cell metabolome and to understand which metabolites were affected. This separation was based on the relationship between metabolites abundance, O_2_ levels and incubation time and that helped in identifying the metabolites that played key roles in the response of oxygen stress. A biplot of scores and loading has presented in Fig. 5. In the new coordinate space, the loadings and scores calculated from the PLS-DA classification model represent the position of the metabolites and the treatments (O_2_ level and time), respectively. Two latent variables (LV's) were chosen for the PLS-DA analysis. The direction of the arrow for the oxygen and incubation time shows how the oxygen treatment increases from 0 kPa through 5 kPa–21 kPa and time from 0 h to 6 h. Metabolites which were grouped together were defined to be favorably connected and exhibit comparable responses in a specific oxygen environment or incubation period, conversely, those that demonstrated opposing reactions to a certain oxygen environment or incubation period were classified as negatively linked. As demonstrated in Fig. 5, the first two LV's might be used to separate the two low oxygen circumstances (5 kPa and 0 kPa) from the control (21 kPa). The percentage of the explained X and Y-variances that could be attributed to these two LV's was 82 and 59, respectively.

The metabolites that were intimately connected to the 0 kPa level included urea, lactate, mannitol, DHAA, pyruvate, ribose, glycerol, glycine, leucine, valine, alanine, serine, threonine, phenylalanine, isoleucine; those associated with 5 kPa included benzoate, sorbitol and trehalose; those associated with the control included galactose, oxaloacetate, oxalate, sorbate, raffinose, cysteine, succinate, 2-keto-gulonate, palmitate, glucuronate, myristate, glycerate, pyroglutamate, phenylactate, quinate, GABA, lyxose, xylose, maleate, isocitrate, β-alanine, glutamine, glutamate, myo-inositol, aspartate, chlorogenate, malate, methionine, stearate, α-KGA, fructose, fumarate, ascorbate, citrate, caffeate, caftarate, shikimate and maltose (Fig. 5). With respect to the time arrow in Fig. 5, even though no metabolites were found to be closely associated with it, when the incubation period rose, the metabolites above the horizontal axis ascended and those below declined. For example, lactate and oxalate, two metabolites which were connected to the low O2 concentrations and the control, in that order, grew over the incubation period while isocitrate and glutamate, connected, respectively, to the control and low oxygen level, were situated across from the time arrowand thus decreased with increasing incubation time.

### 3.5 Changes in amino acids

The most important amino acids that were identified were: alanine, valine, isoleucine, glycine, glutamate, methionine, cysteine, serine, threonine, leucine, aspartate, phenylalanine, glutamine, and the changes in the abundance of these amino acids during incubation has presented in Fig. 4a, Fig. 4b β-alanine, which is a naturally-occurring beta-amino acid; pyroglutamate, a derivative of glutamate and gamma-aminobutyric acid (GABA), which is a significant 4-C amino acid component (not a protein) of the free amino acid pool in most prokaryotes as well as eukaryotes, were also identified. Most of the amino acids either increased or decreased within the 6 h time duration relative to the control. At low O_2_ levels, valine, alanine, aspartate, leucine, glycine, serine, threonine, phenylalanine and isoleucine increased compared to the control, while methionine, glutamate and glutamine decreased relative to the control. Pyroglutamate was also found to be low in abundance in the low O_2_ levels relative to the control. Aspartate showed a fast increase within the first 1 h and then decreased slowly for 0 kPa, but no change was observed for both 5 kPa and control. As like as aspertate, a rapid increase in isoleucine was observed within the first 1 h and decreased slowly up to 5 h for 0 kPa. But, a decreasing circumstance up to 5 h was found for 5 and 21 kPa. After that isoleucine increased for all O_2_ levels. While, alanine and threonine represented a decrease for 21 kPa and increase for 0 kPa and 5 kPa in the first 30 m. Besides, alanine followed an ups and down pattern and threonine showed a continuous decrease with the increasing of time. Glutamine, glutamate increased very rapidly within the first 30 m and pyroglutamate increased sharply within 1 h for the control and then decreased slowly relative to the low oxygen levels. Within 6 h of incubation, an accumulation of leucine and glycine was observed for 0 kPa, while a similar pattern was observed for 5 kPa and control. Serine, phenylalanine and valine increased significantly within the first hour for 0 kPa and then stabilized. For 21 kPa, methionine increased within the first hour, followed by a gradual decrease with incubation time. A different pattern was observed for 5 kPa, where methionine remained stable within the first 5 h and then increased for the 6th hr. For 0 kPa, no change was observed in the pattern of methionine. β-alanine showed a similar pattern between the different oxygen treatments in that it increased within the first 1 h, followed by a gradual decrease with incubation time. GABA increased significantly within the first hour of incubation for 21 kPa and then decreased gradually. Within the first hour of incubation, no change in GABA was observed for 5 and 0 kPa. Under low O_2_ stress a-KGA decreased with time; but, for 21 kPa it was increased for the first 1 h and then, started decreasing up to 5 h.

**Fig. 6:**
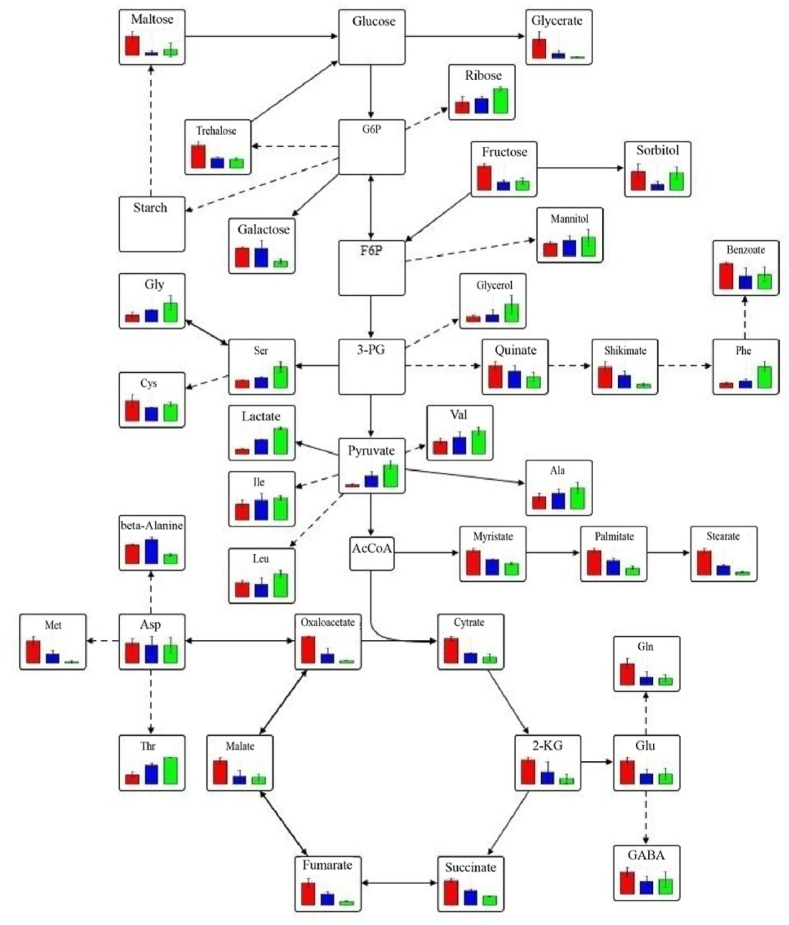
The schematic representation of metabolic pathways (glycolysis, TCA cycle and oxidative pentose phosphate pathway) at time 6 h during O_2_ stress. Solid line in the network indicates a single step connecting two metabolites, and the dotted line represent multiple steps in between. Inside of the blocks showing metabolites at three different O_2_conditions (left side red color: 21 kPa O_2_; middle blue color: 5 kPa O_2_ and right-side green color: 0 kPa O_2_)

From the PLS-DA plot, it was observed that some of the amino acids were positively correlated with the low oxygen (0 and 5 kPa) level, the control and the incubation time, with some being negatively correlated (Fig. 5). A positive correlation of GABA, glutamate, methionine, cysteine, glutamine, and pyroglutamate was observed for 21 kPa, while valine, alanine, leucine, glycine, serine, isoleucine, phenylalanine being negatively correlated with 21 kPa. Also, aspartate, β-alanine, glutamine and glutamic acid showed a negative correlation with an increase in incubation time. These metabolites were mapped on the metabolic networks in Fig. 6 and confirmed by observing the change patterns at the 6 h.

TCA cycle intermediates, α-KGA and oxaloacetate, were the precursors of glutamate and aspartate, respectively [44]. The depletion of glutamate may lead to the down-regulation of Gln and GABA [45]. Serine is also a precursor of glycine. At oxidative stress, alanine, valine, leucine and isoleucine were increased for higher production of pyruvate. Alanine accumulation might indicate the diversion of pyruvate to alanine by reductive amination [46]. Furthermore, Vandendriessche, Schäfer [47] found that the increase in alanine result from enhanced proteolysis provoked by cell death in the brown tissue of the Braeburn apple. GABA and glutamate were also the co-substrate of alanine synthesis, and a decrease in their abundance may be reflected by the increase in alanine levels under low oxygen stress [48]. The increase in threonine levels may be due to increased flux towards its synthesis from aspartate [49], while the levels of β-alanine and methionine were reduced. An elevation in amino acid levels may also be due to the proteolysis of proteins in order to provide indigenous substrates for energy metabolism by the cells [50].

Ampofo-Asiama, Hertog [22] observed a reduction in the abundances of serine, glycine, glutamate, β-alanine and aspartate in cultured tomato cells with oxidative stress but with no change in GABA. In addition, Baxter, Redestig [8] found the depletion in serine, glycine, alanine, aspartate, β-alanine, methionine, threonine, and glutamine in heterotrophic Arabidopsis cells to oxidative stress. Moreover, Oms-Oliu, Hertog [16] also observed a decrease in glutamine, leucine, isoleucine, and serine in the postharvest storage of tomatoes. When Arabidopsis roots were exposed to oxidative stress, Lehmann, Schwarzländer [41] also noticed a decrease in glutamate, aspartate, and methionine and an accumulation in alanine, isoleucine, phenylalanine, and valine content. When conference pears were subjected to low oxygen stress, Pedreschi, Franck [42] marked the increase of GABA through the decarboxylation of glutamate. In addition, Rocha, Licausi [48] noticed that alanine, GABA and glutamate accumulated under hypoxia induced by waterlogging of legumes (*Lotus japonicus*).

### 3.6 Changes in organic acids

The relative metabolic abundance of organic acid with time has shown in Fig. 4a, Fig. 4b. Generally, it was observed that for the low O_2_ concentrations, DHAA, lactate and pyruvate increased within the 6 h of incubation while 2-keto-gulonate, ascorbate, caftarate, caffeate, chlorogenate, citrate, glucuronate, glycerate, maleate, malate, oxalate, oxaloacetate, sorbate, succinate, quinate and shikimate decreased. No significant changes were observed in benzoate between the control and the low oxygen stress within the first 4 h of incubation, followed by an increase in benzoate for the control for the 5 and 6 h of incubation.

Ascorbate increased within the first hour of incubation for the control and then stabilized, but a decreasing trend was observed for two low oxygen levels. For the control, citrate and DHAA did not change during the time course of the incubation, but, on the other hand, it was observed that citrate reduced whereas DHAA increased, respectively, for both 0 kPa O_2_ and 5 kPa O_2_ levels. A gradual increase in the levels of caftarate was observed for the control during the time course of incubation, but no change was observed under low oxygen stress. Under low oxygen stress, lactate increased gradually when compared to the control, whereas glucuronate, caffeate, glycerate, oxalate, oxaloacetate and sorbate decreased within the incubation time. The levels of malate and maleate increased within the first 30 m of incubation and then stabilized for the control, with a decreasing pattern observed when the cells were exposed to oxygen stress. The levels of pyruvate were higher for the 2 oxygen levels within the first 2 h of incubation, and it started accumulating very fast after 3 h for the 0 kPa O_2_ than for the 5 kPa O_2_ level, whereas no change was observed for the control. A gradual decrease was observed for fumarate at low O_2_ levels; but, for control the metabolic rate is first decreasing and then increasing. Shikimate represented a decrease for 0 kPa and increase for 5 kPa and 21 kPa in the first 30 m. Then, the rate is decreased continuously up to 4 h.

From the PLS-DA classification, it can be stated that oxalate, sorbate, succinate, glucuronate, quinate, oxaloacetate, chlorogenate, citrate, malate, maltose, 2-keto-gulonate showed a positive correlation with the control while DHAA, pyruvate, glycerol and lactate showed a positive correlation with 0 kPa oxygen level. Benzoate showed a positive correlation with the 5 kPa oxygen level. On the other hand, isocitrate and glutamate were negatively correlated with the increasing incubation time.

An altered energy metabolism was observed when the tomato cells were incubated under conditions of low oxygen stress. Even though most of the intermediates which were involved in the glycolytic pathway, such as Robison ester (C_6_H_13_O_9_P), neuberg ester (C_6_H_13_O_9_P), and 3-phosphoglycerate, energy metabolism was not identified using the GC-MS approach, probably due to their low levels (below the detection limit of the GC-MS). An accumulation of pyruvate was observed, it is because the accumulation of pyruvate resulted in the inhibition of downstream glycolysis pathways [51]. Other plant systems have shown the buildup of fructose-6-phosphate and glyceraldehyde-3-phosphate under O2 stress [22,52,53] and has been attributed to the need for the plants to increase its glycolytic activity due to the suppression of the TCA cycle. The collection of lactate in avocado fruit as well as in tomato fruit at low oxygen stress conditions was observed by Ke, Yahia [54] and Ampofo-Asiama, Hertog [22] respectively. Pyruvate is used as a substrate for lactate production under anaerobic fermentation. An increase in lactate can be due to anaerobic fermentation and a decrease in TCA cycle activity in low O_2_ environments. The major products of fermentation in plant tissue were ethanol, lactate, and alanine under hypoxia [46].

The depletion of malate was observed in heterotrophic Arabidopsis and conference pears to oxidative stress [8,42]. Ampofo-Asiama, Hertog [22] also observed the same result in cultured tomato fruit cells when they were exposed to oxidative stress. The depletion of malate may notify the perturbation of the TCA cycle [8,46]. Fumarate is the precursor of malate in the citric acid cycle [16] and a decrease in fumarate may lead to a decrease in malate synthesis. In brown pear, the catabolic activity of the malate dehydrogenase enzyme is up-regulated, which decarboxylate malate to pyruvate, causing a decrease in malate and an accumulation in pyruvate [16,47]. Pyruvate is the final product of glycolysis. The accumulation of pyruvate results from the downregulation of its oxidation and an increase in the rate of its synthesis through oxaloacetic acid [46]. Ascorbate reduction was observed by Pedreschi, Franck [42] in conference pears when they were subjected to low oxygen stress. This reduction in ascorbate was due to the downregulation of the ascorbate peroxidase enzyme that impaired the ascorbate glutathione (AsA−GSH) cycle. Ascorbate is a crucial antioxidant compound found in cells. The synthesis of DHAA may be connected to the reduction in ascorbic acid during stressful conditions. When ascorbate breaks down, it means that the AsA−GSH cycle is not able to recycle all of the oxidized ascorbates [8].

Oms-Oliu, Hertog [16] demonstrated a decrease in malate, isocitrate, and succinate and an increase in citrate, 2-keto-gulonate during postharvest storage of tomato. Lehmann, Schwarzländer [41] elucidated that pyruvate rises up and succinate, fumarate, and malate fall down in Arabidopsis roots to oxidative stress. In addition, Franck, Lammerteyn [43] obtained an accumulation of pyruvate, fumarate and depletion of malate, and succinate in storing pears under browning-induced conditions (1 % O_2_ and 10 % CO_2_). Pedreschi, Franck [42] reported the increase of fumarate in conference pears at low oxygen stress, down-regulation of fumarase in brown tissue caused an increase of fumarate and a decrease of malate.

The general decrease in the levels of the organic acids under low O_2_ stress could be employed the survival strategy by the cells to reduce most of their biosynthetic reactions, which consumes energy so as to make substrate available for glycolysis.

### 3.7 Changes in sugars

The major sugars that were identified with the GC-MS in isolated tomato fruit cells included fructose, galactose, lyxose, xylose, arabinose, ribose, trehalose and maltose. The relative metabolic abundance of these sugars has shown in Fig. 4a, Fig. 4b. In general, at low O_2_ stress, it was observed that trehalose, maltose, galactose, lyxose, arabinose and fructose decreased relative to the control, with an increase in ribose. The metabolic abundance of arabinose and xylose increased very rapidly within 30 m of incubation for the control and then decreased slowly. The same pattern was observed for the two low oxygen levels. A high accumulation of galactose was observed within 2 h of incubation for both the control and 5 kPa, with no change in the metabolic abundance observed for the 0 kPa Oxygen level. For the control, lyxose, maltose and trehalose increased very rapidly within the first hour of incubation, but within the last 5 h of incubation, lyxose gradually decreased while maltose and trehalose increased up to the 6th hour of incubation. For the low oxygen level, the metabolic abundance of ribose increased rapidly within the first 30 m of incubation, decreased after 2 h and then remained unchanged, whereas, for the control, it decreased within the 6 h of incubation. In Fig. 5, it was observed that maltose, lyxose, and arabinose were highly correlated with the 21 kPa, while trehalose was highly correlated with the 5 kPa O_2_ level. The decrease in lyxose, arabinose, and fructose might be due to the need for them to be hydrolyzed to be used as a source of energy by the cells; whereas the pentose phosphate pathway (PPP), which is activated and increased activity to maintain the redox balance of cells and create electrons for the reduction of reactive oxygen species, is likely the cause of the rise in ribose.

When oxidative stress was applied to Arabidopsis cells and roots, it was observed that ribose was up-regulated [8,41]. Increased in ribose which is most likely derivatization and degradation products of OPPP intermediates, 6-phosphogluconate and ribose-5-Phosphate, respectively, and the accumulation of this product suggests that there was a relative decrease in glycolytic and TCA intermediates [8]. Moreover, Pedreschi, Franck [42]demonstrated that xylose and trehalose increased in conference pears to oxidative stress, indicating primary cell wall breakdown. However, fructose levels did not change in tomato fruit at low oxygen stress [22]. Galactose was identified as the primary cell wall component, and at low oxygen levels, there was a decrease in galactose and trehalose derived from G6P, indicatinga reduction in cell wall biosynthesis [16].

### 3.8 Changes in sugar alcohols

The important sugar alcohols that were observed in tomato cells by the GC-MS technique were mannitol, sorbitol and myo-inositol. For the low oxygen level (0 kPa), the metabolic response of mannitol increased swiftly within the first 30 min of incubation, gradually decreased for the next 1 h and then remained stable until the end of incubation while the abundance mannitol at 5 kPa oxygen, the level remained constant relative to the control. Within the first 2 h of incubation, it was observed that myo-inositol showed a similar pattern for the three oxygen levels. Within the first hour of incubation, sorbitol was accumulated for the low oxygen level (0 kPa), decreased for the control, and then remained constant within the next 2 h of incubation.

From the PLS-DA biplot, it was confirmed that mannitol was positively correlated with the 0 kPa O_2_ level, while sorbitol was highly correlated with the 5 kPa O_2_ level. Myo-inositol was located in the negative direction of the incubation time (Fig. 5). In the postharvest storage of tomatoes, Oms-Oliu, Hertog [16] noticed that myo-inositol declined with time, and this metabolite was the precursor in the biosynthesis of many cell wall polysaccharides, such as oligosaccharides.

### 3.9 Changes in fatty acids

The relative metabolic abundances in Fig. 4a, Fig. 4b showed that glycerol increased for the low oxygen levels, while stearate, myristate and palmitate decreased at low O_2_ stress. After 4 h of incubation, glycerol increased tremendously for 0 kPa O_2_ level, with a decrease observed for the 5 kPa O_2_ level relative to the control, which remained stable throughout the incubation period of the experiment. The relative metabolic abundances of myristate and palmitate were observed to exhibit increasing patterns for the control oxygen level, while decreasing trends of these metabolites were observed for low oxygen levels, following lower trends at 0 kPa.

In the PLS-DA analysis of Fig. 5, it can be observed that glycerol was correlating with the 0 kPa O_2_ level, while palmitate and myristate were correlating with the 21 kPa O_2_. From the metabolic pathway in Fig. 6, it was observed that glycerol accumulated, whereas myristate and palmitate were reduced at low O_2_ conditions. But Ampofo-Asiama, Hertog [22] mentioned that glycerol does not change oxidative stress on tomato fruit cells.

### 3.10 Changes in other metabolites

It was observed from Fig. 4a, Fig. 4b that urea increased while phenylactate decreased at low O_2_ stress. From the PLS-DA diagrams in Fig. 5, urea correlated g with the 0 kPa O_2_ level. A gradual accumulation of urea was observed for both the 0 kPa O_2_ and 5 kPa O_2_ but decreased slightly after 5 h of incubation for 5 kPa O_2_. At oxidative conditions, protein is utilized for energy production, resulting in the formation of ammonia which ultimately forms urea by the urea cycle. Ampofo-Asiama, Hertog [22] observed that urea did not change in cultured tomato fruit cells during oxidative stress.

The increase in glycerol and urea are probably due to increased flux to their synthesis and the decrease in phenyl actate, myristate and palmitate under low O_2_ stress, maybe a strategy of the cells to hydrolyze them for energy synthesis.

## 4 Conclusions

Tomato, a vegetable crop grown all over the world, is an important and tasty fruit regarding its nutritional and financial worth. To improve the postharvest storage potential of tomatoes under controlled atmosphere conditions, a proper understanding is required of how tomatoes respond and adapt to the stresses imposed upon them during postharvest storage. Tomato cell suspension cultures demonstrated their effectiveness in examining the metabolic impacts of low oxygen stress on tomato cells. This approach unveiled noticeable alterations in the quantities of essential metabolites, highlighting its efficiency as a research tool. The metabolic response provided an interesting idea and helped in figuring out which important regulatory substances were engaged in metabolism. On the basis of metabolic profile, it was proven that oxidative stress significantly inhibits central metabolic processes. A novel concept was revealed by the metabolie analysis of tomato fruit cells to oxygen stress and identified the key regulatory molecules which were involved in metabolism. In the future, fluxes can be determined by steady state labeling experiments to know the impact of oxygen stress on tomato fruit cells' core metabolisms.

## Data availability statement

Data will be made available upon request to corresponding author.

## Compliance with ethical standards

This study does not involve any human or animal testing.

## CRediT authorship contribution statement

**Md. Sultan Mahomud:** Writing – review & editing, Writing – original draft, Validation, Methodology, Investigation, Formal analysis. **Md. Nahidul Islam:** Writing – review & editing, Validation, Formal analysis, Data curation. **Joysree Roy:** Writing – review & editing, Validation, Methodology, Data curation.

## Declaration of competing interest

The authors declare the following financial interests/personal relationships which may be considered as potential competing interests:Md. Sultan Mahomud reports equipment, drugs, or supplies was provided by Laboratory of Mechatronics, Biostatistics and Sensors, KULeuven, Belgium.

## References

[1] Ghosh U.K., Islam M.N., Siddiqui M.N., Khan M.A.R. (2021). Understanding the roles of osmolytes for acclimatizing plants to changing environment: a review of potential mechanism. Plant Signal. Behav..

[2] Ghosh U., Islam M., Siddiqui M., Cao X., Khan M. (2022). Proline, a multifaceted signalling molecule in plant responses to abiotic stress: understanding the physiological mechanisms. Plant Biol..

[3] Noctor G., De Paepe R., Foyer C.H. (2007). Mitochondrial redox biology and homeostasis in plants. Trends Plant Sci..

[4] Van Dongen J.T., Licausi F. (2016).

[5] Sachs M., Vartapetian B. (2007). Plant anaerobic stress I. Metabolic adaptation to oxygen deficiency. Plant Stress.

[6] Drew M.C. (1997). Oxygen deficiency and root metabolism: injury and acclimation under hypoxia and anoxia. Annu. Rev. Plant Physiol. Plant Mol. Biol..

[7] Benkeblia N. (2021). Physiological and biochemical response of tropical fruits to hypoxia/anoxia. Front. Plant Sci..

[8] Baxter C.J., Redestig H., Schauer N., Repsilber D., Patil K.R., Nielsen J., Selbig J., Liu J., Fernie A.R., Sweetlove L.J. (2007). The metabolic response of heterotrophic Arabidopsis cells to oxidative stress. Plant Physiol..

[9] Carrera F.P., Noceda C., Maridueña-Zavala M.G., Cevallos-Cevallos J.M. (2021). Metabolomics, a powerful tool for understanding plant abiotic stress. Agronomy.

[10] Ho P.L., Tran D.T., Hertog M.L.A.T.M., Nicolaï B.M. (2021). Effect of controlled atmosphere storage on the quality attributes and volatile organic compounds profile of dragon fruit (Hylocereus undatus). Postharvest Biol. Technol..

[11] Park D., Al Shoffe Y., Algul B.E., Watkins C.B. (2022). Fermentative metabolism of three apple cultivars during storage under low partial pressures of oxygen. Postharvest Biol. Technol..

[12] Schreinemachers P., Simmons E.B., Wopereis M.C. (2018). Tapping the economic and nutritional power of vegetables. Global Food Secur..

[13] Opeña R., Chen J., Kalb T., Hanson P. (2001).

[14] Quinet M., Angosto T., Yuste-Lisbona F.J., Blanchard-Gros R., Bigot S., Martinez J.-P., Lutts S. (2019). Tomato fruit development and metabolism. Front. Plant Sci..

[15] Ampofo-Asiama J., Baiye V., Hertog M., Waelkens E., Geeraerd A., Nicolai B. (2014). The metabolic response of cultured tomato cells to low oxygen stress. Plant Biol..

[16] Oms-Oliu G., Hertog M.L.A.T.M., Van de Poel B., Ampofo-Asiama J., Geeraerd A.H., Nicolaï B.M. (2011). Metabolic characterization of tomato fruit during preharvest development, ripening, and postharvest shelf-life. Postharvest Biol. Technol..

[17] Wegner L.H., Frey W., Schönwälder S. (2013). A critical evaluation of whole cell patch clamp studies on electroporation using the voltage sensitive dye ANNINE-6. Bioelectrochemistry.

[18] Castoria R., Mannina L., Durán-Patrón R., Maffei F., Sobolev A.P., De Felice D.V., Pinedo-Rivilla C., Ritieni A., Ferracane R., Wright S.A. (2011). Conversion of the mycotoxin patulin to the less toxic desoxypatulinic acid by the biocontrol yeast Rhodosporidium kratochvilovae strain LS11. J. Agric. Food Chem..

[19] Puschmann R., Romani R. (1983). Ethylene production by auxin-deprived, suspension-cultured pear fruit cells in response to auxins, stress, or precursor. Plant Physiol..

[20] Lammertyn J., Franck C., Verlinden B., Nicolaï B. (2001). Comparative study of the O2, CO2 and temperature effect on respiration between ‘Conference’pear cell protoplasts in suspension and intact pears. J. Exp. Bot..

[21] Islam M.N., Wang A., Pedersen J.S., Sørensen J.N., Körner O., Edelenbos M. (2019). Online measurement of temperature and relative humidity as marker tools for quality changes in onion bulbs during storage. PLoS One.

[22] Ampofo-Asiama J., Hertog M.L.A.T.M., Geeraerd A.H., Nicolaï B.M., Waelkens E. (2012). Metabolic profiling of the response of tomato cells to oxygen stress. Acta Hortic..

[23] Roessner U., Wagner C., Kopka J., Trethewey R.N., Willmitzer L. (2000). Simultaneous analysis of metabolites in potato tuber by gas chromatography-mass spectrometry. Plant J..

[24] Wang A., Islam M.N., Johansen A., Haapalainen M., Latvala S., Edelenbos M. (2019). Pathogenic Fusarium oxysporum f. sp. cepae growing inside onion bulbs emits volatile organic compounds that correlate with the extent of infection. Postharvest Biol. Technol..

[25] Xiaohuang C., Azam M., Islam M. (2022). Effect of chitosan and hydroxypropyl starch complex polysaccharide on the physico-chemical properties of Tilapia fish (Oreochromis mossambicus) gel. Food Res..

[26] Nordén B., Broberg P., Lindberg C., Plymoth A. (2005). Analysis and understanding of high‐dimensionality data by means of multivariate data analysis. Chem. Biodivers..

[27] Islam M.N. (2022). Nondestructive Quality Assessment Techniques for Fresh Fruits and Vegetables.

[28] Fernandez-Da Silva R., Menendez-Yuffa A. (2006). Viability in protoplasts and cell suspensions of Coffea arabica cv. Catimor, Elect. J. Biotechnol..

[29] Steward N., Martin R., Engasser J.M., Goergen J.L. (1999). A new methodology for plant cell viability assessment using intracellular esterase activity. Plant Cell Rep..

[30] Smith B.A., Reider M.L., Fletcher J.S. (1982). Relationship between vital staining and subculture growth during the senescence of plant tissue cultures. Plant Physiol..

[31] Kader A.A. (1986).

[32] Lammertyn J., Franck C., Verlinden B.E., Nicolaï B.M. (2001). Comparative study of the O2, CO2 and temperature effect on respiration between ‘Conference’ pear cell protoplasts in suspension and intact pears. J. Exp. Bot..

[33] Solomos T. (1982).

[34] Day D.A., Millar A.H., Wiskich J.T., Whelan J. (1994). Regulation of alternative oxidase activity by pyruvate in soybean mitochondria. Plant Physiol..

[35] Millar A.H., Finnegan P.M., Whelan J., Drevon J.J., Day D.A. (1997). Expression and kinetics of the mitochondrial alternative oxidase in nitrogen-fixing nodules of soybean roots. Plant Cell Environ..

[36] Ribas-Carbo M., Berry J.A., Azcon-Bieto J., Siedow J.N. (1994). The reaction of the plant mitochondrial cyanide-resistant alternative oxidase with oxygen. Biochim. Biophys. Acta Bioenerg..

[37] Ho Q.T., Verboven P., Verlinden B.E., Nicolaï B.M. (2010). A model for gas transport in pear fruit at multiple scales. J. Exp. Bot..

[38] Schauer N. (2004). Metabolic profiling of leaves and fruit of wild species tomato: a survey of the Solanum lycopersicum complex. J. Exp. Bot..

[39] Roessner-Tunali U., Hegemann B., Lytovchenko A., Carrari F., Bruedigam C., Granot D., Fernie A.R. (2003). Metabolic profiling of transgenic tomato plants overexpressing hexokinase reveals that the influence of hexose phosphorylation diminishes during fruit development. Plant Physiol..

[40] Shepherd T., Dobson G., Verrall S.R., Conner S., Griffiths D.W., McNicol J.W., Davies H.V., Stewart D. (2007). Potato metabolomics by GC–MS: what are the limiting factors?. Metabolomics.

[41] Lehmann M., Schwarzländer M., Obata T., Sirikantaramas S., Burow M., Olsen C.E., Tohge T., Fricker M.D., Møller B.L., Fernie A.R., Sweetlove L.J., Laxa M. (2009). The metabolic response of Arabidopsis roots to oxidative stress is distinct from that of heterotrophic cells in culture and highlights a complex relationship between the levels of transcripts, metabolites, and flux. Mol. Plant.

[42] Pedreschi R., Franck C., Lammertyn J., Erban A., Kopka J., Hertog M., Verlinden B., Nicolaï B. (2009). Metabolic profiling of ‘Conference’ pears under low oxygen stress. Postharvest Biol. Technol..

[43] Franck C., Lammerteyn J., Nicolaï B. (2005). Metabolic profiling using GC-MS to study biochemical changes during long-term storage of pears. Acta Hortic..

[44] Khan I., Qayyum S., Maqbool F., Hayat A., Farooqui M.S. (2017).

[45] Guerriero R.M., Giza C.C., Rotenberg A. (2015). Glutamate and GABA imbalance following traumatic brain injury. Curr. Neurol. Neurosci. Rep..

[46] Sousa C.A.F.d., Sodek L. (2002). The metabolic response of plants to oxygen deficiency. Braz. J. Plant Physiol..

[47] Vandendriessche T., Schäfer H., Verlinden B.E., Humpfer E., Hertog M.L.A.T.M., Nicolaï B.M. (2013). High-throughput NMR based metabolic profiling of Braeburn apple in relation to internal browning. Postharvest Biol. Technol..

[48] Rocha M., Licausi F., Araújo W.L., Nunes-Nesi A., Sodek L., Fernie A.R., van Dongen J.T. (2010). Glycolysis and the tricarboxylic acid cycle are linked by alanine aminotransferase during hypoxia induced by waterlogging of Lotus japonicus. Plant Physiol..

[49] Zhang Y., Meng Q., Ma H., Liu Y., Cao G., Zhang X., Zheng P., Sun J., Zhang D., Jiang W. (2015). Determination of key enzymes for threonine synthesis through in vitro metabolic pathway analysis. Microb. Cell Factories.

[50] Dasuri K., Zhang L., Keller J.N. (2013). Oxidative stress, neurodegeneration, and the balance of protein degradation and protein synthesis. Free Radic. Biol. Med..

[51] Chandel N.S. (2021). Glycolysis. Cold Spring Harbor Perspect. Biol..

[52] Mullarky E., Cantley L.C. (2015).

[53] McGarry T., Biniecka M., Veale D.J., Fearon U. (2018). Hypoxia, oxidative stress and inflammation. Free Radic. Biol. Med..

[54] Ke D., Yahia E., Hess B., Zhou L., Kader A.A. (1995). Regulation of fermentative metabolism in avocado fruit under oxygen and carbon dioxide stresses. J. Am. Soc. Hortic. Sci..

